# A critical look at the national essential medicines list of India 2003

**DOI:** 10.4103/0976-500X.72347

**Published:** 2010

**Authors:** S Manikandan

**Affiliations:** *Asst Editor, JPP*

Essential medicines are those that satisfy the priority healthcare needs of the population and are available at all times in adequate amounts, in the appropriate dosage forms with assured quality and at a price the individual and community can afford. Essential medicines are selected with due regard to disease prevalence, efficacy, safety and cost effectiveness. In most developing countries, the amount spent for medicines is the largest public health expenditure on health after personal costs and the largest household health expenditure.[1] In 1977, World Health Organization (WHO) published the first model list of essential drugs. Since then, the remarkable impact of essential medicines in improving rational drug use has been proved in many countries.

The essential medicines list (EML) of the WHO is called a “model list” as it is a guide for the development of national essential medicines list (NEML) which in turn should help in forming the institutional list and P drugs. The concept of essential medicines is global and more than 150 countries have their national list. India too has an NEML[2] which was prepared in 2003. A critical analysis of the NEML (2003) was done to review the list in relation to the WHO guidelines for the selection of essential medicines and find out the extent of compliance. Unfortunately, this process led to the discovery of a large number of errors in this national document. The following commentary lists the issues in relation to the errors found in NEML 2003.

## BREAKING THE CONVENTION

Standard conventions are followed in scientific writing, to establish uniformity in presentation to enable comparisons, document changes and trace the temporal history of important documents. Unfortunately, the NEML 2003 flouts many such conventions, such as:

- Every EML should have an edition number. For example, the current WHO EML is the 16th updated list (edition) and the children’s EML (EMLc) is the second edition.[3,4] There is no edition number mentioned in the NEML.
- By convention, the strength of liquid oral dosage forms are given per 5 mL. This is because patients are advised to consume liquid oral dosage forms in teaspoons and one teaspoon measures 5 mL. NEML does not follow this convention and it gives the strength of syrups in a wrong manner, e.g., syrup phenytoin.
- Bupivacaine is mentioned in the NEML as “injection 0.5% + 7.5% glucose.” Conventionally, fixed dose drug combinations alone are written in this manner. This might mislead the reader and it should be clearly mentioned that both have to be mixed separately and this should be used only for spinal anesthesia. This has been done in the WHO EML.

## SURPLUS IS NOT ESSENTIAL

In the selection of essential medicines, relative cost-effectiveness is a major consideration for choosing drugs within the same group. NEML includes pheniramine maleate, chlorpheniramine maleate and dexchlorpheniramine maleate as antihistaminics. Even though these three different drugs are available in three different formulations, all the three need not be included in the essential list as these three medicines do not differ in efficacy but only in their pharmacokinetic characteristics. Chlorpheniramine maleate is listed in three formulations (tablet, syrup and injection) and this alone need be included.

Drugs from the same group which do not differ much from the prototype need not be included in an essential list. Many antibiotics from the same class are included in the NEML. For example, four macrolide antibiotics (erythromycin, roxithromycin, clarithromycin and azithromycin) have been included, as in the case of cephalosporins. Clearly, the basic considerations of efficacy, safety, suitability and cost have not been rigorously applied.

Even though the NEML lists eleven antiarrhythmics, the commonly used ones (e.g. lignocaine) are not listed.

## IMPROPER SELECTION

WHO clearly defines the selection criteria for essential medicines and also explains the method to develop a national list and its implementation. There is evidence that these have been overlooked while developing the NEML such as:

Drugs which form the first-line treatment for certain infections are not included whereas second-line drugs have been included. For example, a single dose of praziquantel results in nearly 100% cure rate for tapeworm infestations. Niclosamide is usually used only if praziquantel fails. The NEML includes only niclosamide.

Drugs for which best evidence for effectiveness and safety exist are not included while some other drugs in the same group are added. For example, atenolol has the best evidence among many β-blockers, yet it is not included as an antianginal. Permethrin is the drug of choice for scabies and both the WHO EML and EMLc list it. However, the NEML has listed gamma benzene hexachloride which is potentially neurotoxic in children.

Even though both amoxicillin and ampicillin are included in WHO EML and EMLc, the formulations differ. Amoxicillin is listed for oral use and ampicillin parenterally. However, National EML lists oral formulations for amoxicillin and ampicillin. Ampicillin is inferior to amoxicillin by oral route as it has less oral bioavailability and high incidence of diarrhoea. Oral formulations of ampicillin need to be deleted from National EML.

## SIGNIFICANT OMISSIONS

Omitting an essential drug can have a significant effect on the access to medicines. The NEML has omitted some important essential drugs such as:

- Iron and folic acid tablets do not form a part of the NEML. Considering the high prevalence of anemia in pregnant women in India, iron and folic acid supplementation during pregnancy is very important. The fixed dose combination of ferrous sulfate + folic acid should be made available in all primary health centres (PHCs) so that every pregnant woman has access to it. The WHO EML includes it, but not the NEML.
- Many drugs (paracetamol, morphine, steroids such as dexamethasone, hydrocortisone, prednisolone, many antibiotics, diuretics such as furosemide, hydrochloro-thiazide, spironolactone and other drugs such as diazepam, warfarin, phenytoin, etc.) do not have either the dose or dosage form appropriate for children.
- NEML lists pralidoxime chloride as an essential medicine at secondary and tertiary levels of health care. Insecticide poisoning is more common among villages and hence more cases will be encountered at PHCs. The list should change pralidoxime as an essential medicine at all levels especially since the distances to transport patients to tertiary care centers may be long and patients may not reach these centers in time for pralidoxime to be effective.
- The elimination of phenytoin changes from first order to zero order above the dose of 300 mg/day. Titration of dose above this limit should be in the magnitude of 25 mg. Hence, 25 mg tablets are not only needed for children but also for adults and have therefore been included in WHO EML and EMLc. The NEML does not list phenytoin tablets of this strength.
- No fixed dose combination of first-line antitubercular drugs has been included in the NEML. The combination of four drugs (isoniazid + rifampicin + ethambutol + pyrazinamide) and two drugs (isoniazid + rifampicin) are essential and should be included. No second-line antitubercular agent (except ofloxacin) is included in the NEML. They are essential for secondary and tertiary care hospitals.
- No drug other than chloroquine is included for prophylaxis of malaria.
- Lipid lowering drugs are conspicuous by their absence.
- The NEML includes bleaching powder as one of the disinfectants. However, there is no mention about the amount of bleaching powder that should be made into solution or the percentage of available chlorine. These details need to be included. Similarly, formaldehyde solution, though listed does not contain the necessary information about the strength of the solution (percentage).

## ERRORS

Spelling mistakes, especially of the names of drugs which are usually blamed on the hapless typist are plentiful in this 48 page document. Notable among them are “odansetron”, “ethnyl estradiol”, “ceftadizidime” and “riboflavine”. It is a sad example that a document which has many of the top pharmacologists of the country as contributors has not been proof read adequately to ensure the correct spelling of drug names.

More serious and dangerous issues are the errors in amount (strength) of drug in the dosage form. The strength of syrup phenytoin sodium is wrongly mentioned as “25 mg/ml”. It should be 5 mg/ml or 25 mg/5 ml. Similar errors exist for metoclopramide and ritonavir. The dose of inhaled beclomethasone is erroneously mentioned as 250 mg instead of 250 µg. Same is the case for salbutamol and anti-D immunoglobulin injection. As we are all aware of the fact that errors in the strength can be dangerous, they can either cause lack of efficacy or toxic effects which may at times be fatal.

The errors in dose do not end here. The strength of antacid tablets and suspension is omitted. The strength of neomycin + bacitracin ointment is mentioned as “5 mg + 500 IU”. It is not mentioned whether it is per gram or per milligram of ointment. The constituents of oral rehydration salt (ORS) are just mentioned “as per I.P”. All the constituents should be clearly listed as there are many ORS preparations—low osmolality ORS, rice-based ORS, high osmolality (old) ORS, etc.

Factual errors are also a plenty in the national list. Atropine sulfate is listed as a nonspecific antidote. Atropine is a specific antidote for insecticide (organophosphorus) poisoning and cannot be used as an antidote for any other poison. Menadione is not preferred for children. Even though WHO EML and EMLc do not include it, the NEML includes it. The composition of procaine penicillin is erroneously listed as “Crystalline penicillin (1 lac units) + procaine penicillin (3 lac units)”. Actually, procaine penicillin is an aqueous suspension of crystalline penicillin (3 lac units) and procaine (120 mg). Even though there is a separate section for beta lactam antibiotics, cephalexin is mentioned under “other antibiotics”.

## THE WAY AHEAD

Even though the expert committee has mentioned that the NEML will be reviewed every 2 years like the WHO EML, more than 7 years have elapsed since the last list. Many mistakes and improper selection of drugs in the list make one wonder whether adequate effort and care were taken in the preparation of this important document. It is hoped that when the list is revised, due considerations are given to the points raised in this communication.
